# Extreme heat disproportionately increases severe road traffic crashes in high conflict settings and among vulnerable road users in California

**DOI:** 10.1007/s44327-026-00248-6

**Published:** 2026-04-22

**Authors:** Cheng-Kai Hsu, D. Alex Quistberg, Carolina Pérez-Ferrer, Daniel A. Rodríguez

**Affiliations:** 1 Department of City and Regional Planning and Institute of Transportation Studies, University of California, Berkeley, CA USA; 2 Dornsife School of Public Health, Drexel University, Philadelphia, PA USA; 3 Center for Research in Population Health, National Institute of Public Health, Cuernavaca, Morelos Mexico; 4 Institute of Transportation Studies, University of California, Berkeley 107C McLaughlin Hall, MC 1720 , Berkeley, CA 94720 USA

**Keywords:** Road traffic crash, Extreme heat, Bicyclist, Pedestrian, Violation, Severity

## Abstract

Emerging evidence suggests that extreme heat elevates road-traffic injuries, undermining international road safety efforts like Vision Zero as global warming intensifies. However, the mechanisms underlying heat-related crashes remain poorly understood, with limited research linking heat exposure to specific crash types that may be driven by heat-induced unsafe road behaviors. Here, we analyzed temperature data and police-reported crash records—including detailed crash scene information—from 177 California cities (2012–2023) using a time-stratified case-crossover design, examining variations in risk by crash type and contextual factors. We observed that extreme heat was associated with elevated risks of fatal and severe crashes, with risk increasing monotonically at higher temperatures. Associations were more pronounced in high-conflict settings such as intersections; among vulnerable road users, including bicyclists and pedestrians; and in crashes involving driver-related violations common to these contexts, such as improper turning and failure to yield to pedestrians, and instances where pedestrians or bicyclists were themselves the at-fault parties. These patterns are consistent with the possibility that extreme heat may increase crash risk by influencing road behavior, reinforcing behavioral mechanisms as a plausible pathway. They also highlight potential disparities in road safety, particularly affecting individuals who continue to walk or cycle and remain mobile during extreme heat, such as low-income individuals without access to climate-controlled vehicles. Urban planning and transportation policies should integrate climate resilience into road safety strategies, such as shaded intersections, cooling interventions for vulnerable road users, enhanced right-of-way protections during extreme heat, and institutional recognition of heat as a situational risk factor.

## Introduction

Road-traffic injuries are a major health concern worldwide, prompting international initiatives such as Vision Zero, yet global warming increasingly threatens progress toward these goals by intensifying heat-related crash risks. A global analysis of Global Burden of Disease (GBD) data (1990–2019) found that while age-adjusted road traffic mortality rates declined over time, the number of heat-related road deaths increased from 20,300 in 1990 to 28,400 in 2019, highlighting that heat-related road safety burdens may rise even as overall traffic safety improves [1]. As global warming leads to more frequent, intense, and prolonged periods of higher temperatures [2–4], with projected end-of-century warming of roughly 4–5 °C and a 13- to 30-fold increase in population exposure to extreme heat across US cities, addressing heat-related road injuries is critical in advancing global road safety goals.

Growing evidence further supports that high ambient temperatures increase the risk of road-traffic injuries, particularly among vulnerable road users such as pedestrians and bicyclists. While the health impacts of heat on natural-cause mortality are well established [5–7], recent studies show that heat also contributes to unintentional injuries [8, 9], including road-traffic fatalities [10]. A meta-analysis of 16 studies, mostly from temperate to warm climates, found that each 1 °C temperature rise increases injury risk by 1.086 (95% CI: 1.064, 1.108) [11]. In the U.S., an analysis of National Highway Traffic Safety Administration (NHTSA) fatal crash records (2001–2011) found a 3.4% rise in daily fatal crashes on heat-wave days (defined as periods when the daily mean temperature exceeded the local 95th percentile for at least two consecutive days) [12]. Further studies show that these risks are even higher for bicyclists and pedestrians who have direct exposure to outdoor heat and high vulnerability to severe crash outcomes [13, 14].

These concerns are particularly pressing in places such as California, USA, where rising temperatures coincide with increasing road fatalities involving vulnerable road users. In 2022, California accounted for over 10% of all U.S. road deaths, including 15% of pedestrian fatalities and 16% of bicyclist fatalities [15]. Fatal and severe crashes in California have risen since 2010 [16], with a 28% rise between 2019 and 2022, outpacing the national average and reaching a decades-long high [17]. Meanwhile, many California cities face growing climate threats, with 40–53 extreme heat days expected annually by 2050, rising to 40–99 by 2099 [18].

However, few studies have directly investigated how extreme heat impacts specific crash types or traffic violations, an approach that can clarify underlying mechanisms and inform more actionable mitigation strategies. Most existing studies have focused on overall statistical associations between heat and crash outcomes, with interpretations relying on indirect or external evidence. For instance, several time-series and case-crossover studies have shown that higher ambient temperatures increase the risk of motor vehicle crashes [12, 19–22]. Yet these analyses generally remain at the aggregate level and do not disaggregate by crash characteristics such as crash type, traffic violation, or at-fault party. As a result, interpretations often rely on indirect or external evidence. For example, mechanically, heat may lead to vehicle failures such as engine overheating and tire blowouts [23]. For infrastructure, heat may degrade road surfaces, causing pavement buckling and asphalt softening [24]. Behaviorally, heat can lead to driver fatigue [25, 26], reduced attentiveness [27], and increased aggression and risky driving [28], a dangerous mix that may manifest in higher crash and injury numbers. Examining effect modification—such as whether heat disproportionately increases the risk of certain types of crashes—can better clarify these dynamics.

Here we aim to deepen the understanding of the mechanisms underlying heat-related road crashes by analyzing police-reported crash data from 177 California cities (2012–2023), with a focus on variations by crash type and contextual crash factors such as severity, modal involvement, occurrence setting, and at-fault factors. To our knowledge, no prior study has examined crash-level variations in heat-related road incidents across such a large sample of cities. A strength of our study is the use of police-reported crash records, offering greater detail than commonly used sources like death certificates or hospitalization records. This enables a more nuanced analysis of crash circumstances and risk progression than previous research typically focused on aggregate crash counts and broad risk estimates. Our multi-city dataset also offers the statistical power needed to examine road crashes, an area often underexplored due to the rarity of such events and the substantial data required for robust analysis.

## Methods

California’s climate is diverse, ranging from hot desert in the southeast to alpine tundra in the Sierra Nevada. Coastal regions, the Central Valley, and the Sierra Nevada foothills are characterized by a Mediterranean climate [29]. Proximity to the Pacific Ocean moderates extremes in coastal areas, producing cooler summers and milder winters, whereas inland valleys and desert regions experience hotter summers and greater diurnal and seasonal variability. This climatic heterogeneity provides a diverse context for examining heat-related road safety risks.

We collected road-traffic crash data from 2012 to 2023 for 177 California cities with population over 50,000 as of 2019. We excluded 2020 to reduce bias from the disruptions in mobility and crash reporting during the onset of the COVID-19 pandemic. Although some pandemic-related effects may have persisted into 2021–2022, statewide vehicle miles traveled (VMT) had largely rebounded by mid-2021 and exceeded 2019 volumes by 2022 [30], supporting our decision to exclude only 2020. Road-traffic crash data were sourced from the Statewide Integrated Traffic Records System (SWITRS) [31], which compiles all crashes reported to the California Highway Patrol and is cleaned, geocoded, and typed by UC Berkeley’s Safe Transportation Research & Education Center (SafeTREC) [32]. Daily temperature and precipitation data at 800-meter spatial resolution were sourced from the PRISM Climate Group [33] and population-weighted per year using U.S. Census Tract Population Data from the National Institutes of Health [34]. Due to data availability constraints, population figures from 2020 were used for weighting between 2021 and 2023.

The data were structured as a daily time series, with daily crash counts as the outcome and daily mean temperature (Tmean, °C) as the primary exposure. Daily precipitation (inch) was included as a covariate. For selected stratified analyses, daily maximum (Tmax) and minimum (Tmin) temperatures were used as alternative exposure variables, as described below. Crashes were categorized by seven stratification factors to examine variation in risk: collision severity, modal involvement, crash type, intersection occurrence, crash time, reported violations, and the at-fault party’s transportat mode.

1. Collision severity was categorized into three levels, following the definitions used in the SWITRS and consistent with the Model Minimum Uniform Crash Criteria [35]: (1) fatal injury, defined as any crash in which at least one person died within 30 days of the collision; (2) severe injury, including visible injuries requiring hospitalization such as major lacerations, broken or distorted extremities, suspected skull/chest/abdominal injuries, significant burns, unconsciousness, or paralysis; and (3) minor injury, which includes minor injury, possible injury, such as abrasions, minor lacerations, or complaints of pain without visible wounds.
2. Modal involvement was classified based on the first object struck in the crash, including six categories: (1) pedestrian, (2) bicyclist, (3) other motor vehicle on the same roadway, (4) other motor vehicle on other roadway, (5) fixed object, and (6) others/not stated.
3. Crash types were classified into eight subcategories (head-on, sideswipe, rear-end, broadside, hit-object, overturned, vehicle-to-pedestrian, and others) and further grouped them into three broader categories: (1) multi-vehicle (head-on, sideswipe, rear-end, and broadside), (2) single-vehicle (hit-object and overturned), and (3) vehicle-pedestrian, to capture both detailed and overarching patterns.
4. Crashes were classified by whether they occurred at an intersection, including three categories: (1) yes, (2) no, or (3) not stated.
5. Crash time was classified using two approaches. In the primary specification, crash timing was defined using city-specific sunrise and sunset times matched to each crash date and location (solar-time classification) [36], classifying crashes as daytime (between sunrise and sunset) or nighttime (between sunset and the following sunrise). For this analysis, Tmax was used for daytime crashes and Tmin for nighttime crashes to better reflect time-specific heat exposure. As a sensitivity analysis, crash time was alternatively classified based on the reported clock time of occurrence, dichotomized into daytime (06:00–17:59) and nighttime (18:00–05:59) [37].
6. For reported violations, there were over 20 original subcategories (e.g., unsafe speed, improper turning, pedestrian right-of-way violation, see Table S2 for the full list). We grouped them into three broader categories: (1) driver-related violation, (2) pedestrian violation, and (3) others.
7. Transport modes used by the at-fault party included four broad categories: (1) pedestrian, (2) bicycle, (3) motor vehicle, or (4) other/not stated.

We calculated descriptive summary statistics of city-level averages of annual crash rate (per 100,000 population) and mean temperatures. These metrics were then summarized across all cities, overall and stratified by the aforementioned factors. In addition, we quantified the proportion of city-days with temperatures at or above the 99th percentile and the proportion of crashes occurring on those days, stratified by crash severity, to provide descriptive context for extreme heat exposure.

We applied a time-stratified case-crossover design, a robust and increasingly used approach for assessing short-term effects of environmental exposures on transient health outcomes [38, 39], including road-traffic injuries [19]. In this design, each case day was matched to control days that fell on the same day of the week within the same month and year (using a 1:3 or 1:4 matching scheme, depending on the number of weekdays per month). By design, the case-crossover approach differs from conventional time-series regression, which models population-level exposure–response associations across the entire study period. Instead, it compares each case with its own matched controls, thereby inherently adjusting for individual-level, time-invariant confounders (e.g., demographic characteristics) and controlling for broader temporal factors such as long-term trends, seasonality, and day-of-week effects. Importantly, this matching strategy also indirectly accounts for variation in traffic volume, since comparisons are restricted to days with similar baseline travel patterns—for example, Mondays are compared only with other Mondays within the same city and month. Following prior work [19, 40], we thus rely on day-of-week matching as an established method to capture routine traffic volume patterns while still allowing for the possibility that heat-related changes in travel behavior contribute to the observed associations.

To conduct the multi-city analysis, we applied a one-step modeling approach, pooling data from all cities into a single framework [41, 42]. The alternative two-step method (i.e., estimating city-specific models and then combining results via random-effects meta-regression) is commonly used in multi-city time-series studies, but it is less suitable in our context. As fatal and severe crashes are relatively rare, many smaller cities had sparse data, which can lead to unstable estimates or model convergence problems when fitted separately [42]. The one-step approach overcomes this limitation by jointly modeling all cities, with city incorporated into a four-way strata term (city–year–month–day of week). This ensures that each case city-day is matched only with control city-days within the same city, calendar month, and weekday, thereby preserving statistical power, stabilizing estimation, and allowing a robust multi-city analysis despite data sparsity.

We estimated temperature-crash associations using a distributed lag non-linear model (DLNM) within a conditional quasi-Poisson framework [43]. The DLNM captures both exposure-response and lag-response relationships using a bi-dimensional cross-basis term. We placed three knots at the 10th, 75th, and 90th temperature percentiles, following established model specifications [44] and their application in road-traffic injury research [45], and specified a two-day lag, consistent with prior work showing that heat-related crash risks peak within this timeframe in U.S. metropolitan areas [20]. The model can be formally expressed as follows:

$$\:{\mathrm{Y}}_{\mathrm{it}}\sim \mathrm{quasiPoisson}\left({{\upmu\:}}_{\mathrm{it}}\right),$$

$$\mathrm{log}\left({{\upmu\:}}_{\mathrm{it}}\right)=\alpha\:+\mathrm{cb}\left({\mathrm{T}}_{\mathrm{it}},l\right)+\beta\:{\mathrm{P}}_{\mathrm{it}}+\mathrm{strata}\left(i, y, m, dow\right),$$

where 𝑌_𝑖𝑡_​ denotes the number of crashes in city 𝑖 on day 𝑡, and 𝜇_𝑖𝑡_​ is the corresponding expected number of crashes. The term $\:cb({T}_{it},l)$ is the cross-basis function for temperature $\:{T}_{it},$ across lag $\:l$, and $\:{P}_{it}$ represents daily precipitation. The strata term denotes matching by city, year, month, and day of week. The cross-basis function was parameterized using natural cubic splines, following the standard DLNM approach [43]:

$$\mathrm{cb}\left({\mathrm{T}}_{\mathrm{it}},\mathrm{l}\right)=\sum_{\mathrm{k}=1}^{{\mathrm{K}}_{\mathrm{T}}}\sum_{\mathrm{m}=1}^{{\mathrm{K}}_{\mathrm{L}}}{\theta\:}_{\mathrm{km}}{\mathrm{B}}_{\mathrm{k}}\left({\mathrm{T}}_{\mathrm{it}}\right){\mathrm{L}}_{\mathrm{m}}\left(l\right),$$

where Bk​​(⋅) are spline basis functions for the exposure–response dimension (with knots at the specified percentiles of the city-specific temperature distribution) and Lm​​(⋅) are spline basis functions for the lag dimension. The tensor product of these two sets of basis functions allows the DLNM to estimate a smooth two-dimensional surface of effects across both temperature and lag, while the coefficients θkm ​quantify the contribution of each temperature–lag basis combination. After fitting the cross-basis model, cumulative log-relative risks were calculated by summing the estimated temperature–lag contributions across the specified lag period. The cumulative relative risk at each temperature was then obtained by exponentiating the summed log-relative risk.

We began by estimating the main effects, capturing the overall association of temperature with crashes of all types. We then conducted stratified analyses across the seven stratification variables described above, starting with collision severity. For the remaining six stratification variables, we followed two analytical steps: (1) analyzing all crashes combined and (2) then analyzing fatal and severe crashes separately, to assess whether associations varied by severity. The overall analytical framework is illustrated in Fig. S1. To ensure model stability, we reported results only for strata with at least five crashes per 100 city-days.

Relative risks (RRs) at the 99th temperature percentile were estimated with 95% CIs, relative to the minimum crash risk temperature—the temperature percentile associated with the lowest RR between the 1st and 99th percentiles in the main effects. To assess the impact of extreme heat, we followed an established approach used by Kephart et al. [46] and derived the log(RR) at the 99th compared with the 95th percentile of the observed distribution of daily temperatures, dividing it by the difference in °C between the 99th and 95th percentiles of the temperature distribution. The derived RR slope represents the percent change in log(RR) per 1 °C increase in temperature between the 95th and 99th percentiles and quantifies how rapidly risk increases at the extreme end of the temperature distribution. Additionally, attributable fractions (AFs) were estimated for days with temperatures at or above the 99th percentile, representing the proportion of crashes occurring on extreme-heat days that may be attributable to elevated temperatures relative to the minimum crash risk temperature under the model assumptions. All analyses were conducted in R (version 4.3.1). Modeling was performed using the dlnm (version 2.4.7), gnm (version 1.1.5), and splines (version 4.3.1) packages.

## Results

### Data description

In total, 1,411,598 road-traffic crashes were observed over 711,009 city-days and available for analysis. Fig. S2 shows the location of each city along with city-level averages of temperature conditions and road crash rates.

Table S1 summarizes overall crash statistics, and Table S2 offers detailed breakdowns by crash type and violation category. On average, cities experienced 1.09 crashes per 100,000 population per day (SD = 0.36; median = 1.10; range = 0.31–1.99). By severity, fatal crashes occurred at a much lower rate (mean = 0.02), while severe and minor injury crashes averaged 0.06 and 1.02, respectively. By crash type, multi-vehicle crashes were most common, particularly rear-end and broadside (T-bone) collisions, followed by single-vehicle and vehicle-pedestrian crashes. By modal involvement, crashes involving occupants of other motor vehicles were most frequent, followed by those involving fixed objects, bicyclists, pedestrians, and motor vehicles on other roadways. Crashes occurred more often at non-intersections than intersections, and were more common during the day than at night. Driver-related violations were more frequent than pedestrian violations, with unsafe speed, automobile right-of-way violations, and improper turning being the most common subcategories. Motorists were most often at fault, followed by bicyclists and pedestrians. The average daily temperature was 17.5 °C (SD = 1.71; range = 13.6–23.9 °C), and average precipitation was 1.00 inches (SD = 0.40; range = 0.19–2.62 inches).

Across all cities, roughly 1.00% of city-days met the ≥ 99th percentile temperature threshold. On those days, 1.02% of all crashes occurred, including 1.01% of minor crashes, 1.10% of severe crashes, and 1.12% of fatal crashes, showing a progressively greater concentration of crashes with increasing severity relative to the frequency of extreme heat days.

### Associations between temperature conditions and road-traffic crashes

**Fig. 1:**
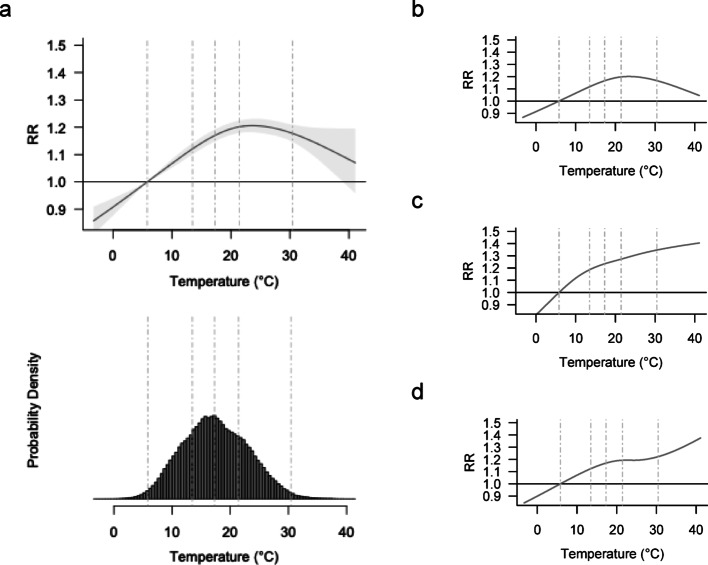
Temperature-crash associations, overall and by collision severity. **a**, overall; **b**, minor injury; **c**, severe injury; **d**, fatal injury. Dashed lines indicate the 1st, 25th, 50th, 75th, and 99th percentiles of the temperature distribution

Figure 1a shows the overall temperature-crash association, representing the main effects of temperature on crash risks without stratification. Overall, risk followed an inverted U-shape, peaking near the 80th percentile and declining thereafter. The minimum crash risk temperature was at the 1st temperature percentile (5.8 °C), suggesting that the RR was lowest at the coldest observed temperatures. Compared to this reference, the RR at the 99th temperature percentile (30.4 °C) was 1.18 [95% CI: 1.15, 1.21]. The RR slope, defined as the percent change in log(RR) per 1 °C increase between the 95th and 99th percentiles, was − 0.50%/°C, indicating a decline in risk at the upper extreme. The estimated AF for temperatures at or above the 99th percentile was 0.08% [0.07%, 0.10%].

Temperature-crash associations for minor crashes (Fig. 1b) mirrored the main effects, likely reflecting its dominance in the dataset. In contrast, severe (Fig. 1c) and fatal crashes (Fig. 1d) showed continued risk increases at higher temperatures without a subsequent decline at the hottest end. RRs at the 99th percentile were 1.17 [1.14, 1.20] for minor crashes, 1.35 [1.20, 1.51] for severe crashes, and 1.22 [1.01, 1.49] for fatal crashes. Corresponding RR slopes were − 0.59, 0.54, and 0.55%/°C, respectively, indicating sharper risk increases at extreme heat levels for more severe outcomes. The estimated AFs for temperatures at or above the 99th percentile were 0.07% [0.06%, 0.10%] for minor crashes, 0.17% [0.10%, 0.25%] for severe crashes, and 0.14% [− 0.12%, 0.37%] for fatal crashes.

Table 1 summarizes estimated RRs at the 99th temperature percentile, RR slopes between the 95th and 99th percentiles, and AFs at or above the 99th percentile from all stratified analyses, including both all-severity and fatal/severe crashes. Key findings are detailed below.

**Fig. 2:**
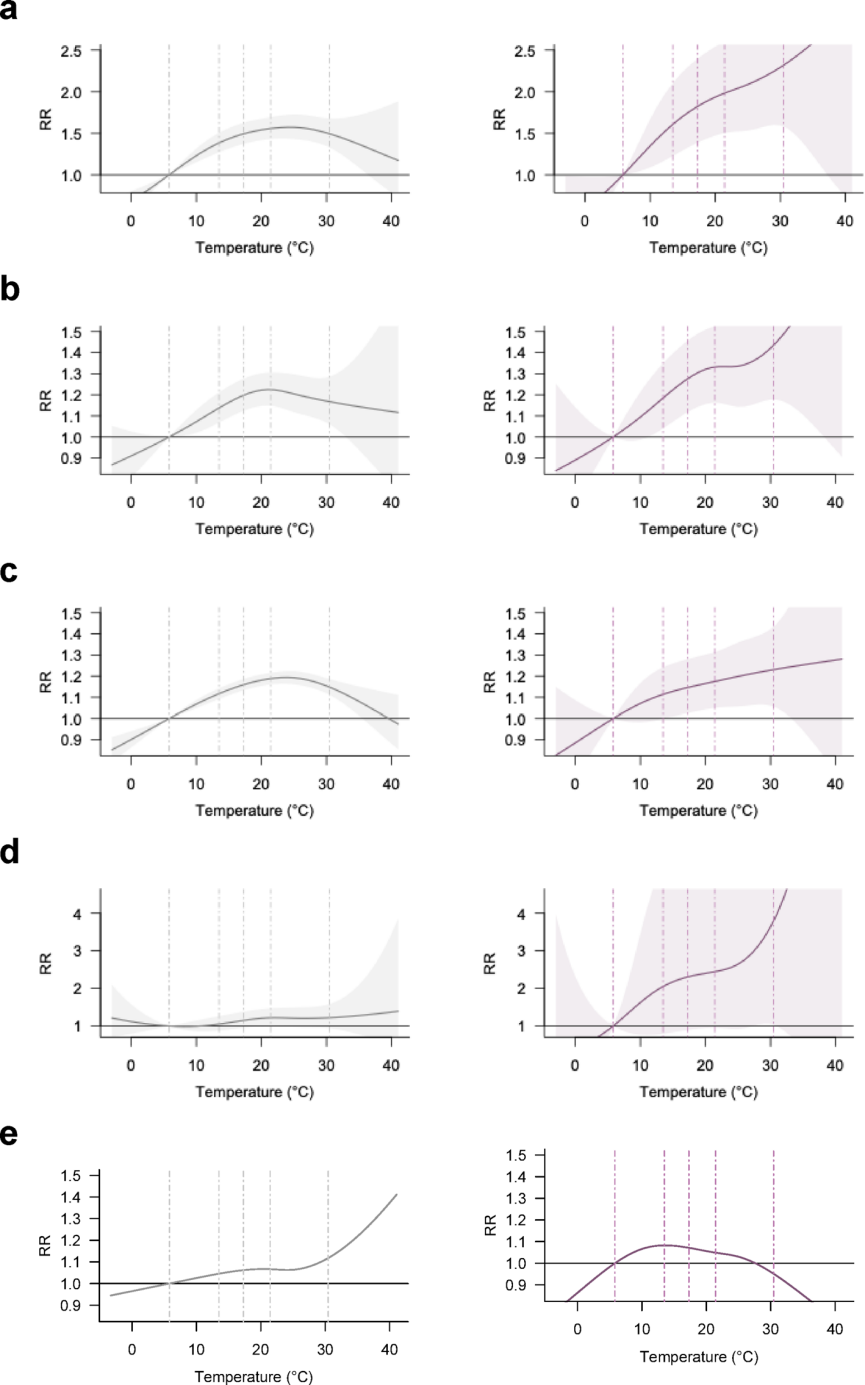
Temperature-crash associations by modal involvement. **a**, bicycle; **b**, pedestrian; **c**, motor vehicle on the same roadway; **d**, motor vehicle on other roadway; **e**, fixed objects. Left and right panel represent all-severity and fatal and severe crash-specific analysis, respectively. Note that the y-axis scales for subfigures a and d are adjusted to accommodate full curves. Dashed lines indicate the 1st, 25th, 50th, 75th, and 99th percentiles of the temperature distribution

First, modal stratification revealed clear heterogeneity in temperature–crash associations. In all-severity analysis (Fig. 2, left), crashes involving occupants of motor vehicles on the same roadway, bicyclists, and pedestrians followed an inverted U-shaped pattern, with the strongest effects observed for bicyclists (note the different y-axis scale), while fixed-object crashes showed a continuous rise. In fatal/severe crashes (right), all categories except fixed-object crashes showed monotonic increases in risk, though effect sizes varied. Crashes involving motor vehicles on other roadways showed the highest RR (3.77 [1.04, 13.61]), followed by bicyclists (RR = 2.31 [1.59, 3.38]), pedestrians (RR = 1.43 [1.18, 1.74]), motor vehicles on the same roadway (RR = 1.23 [1.06, 1.43]), and fixed objects (RR = 0.95 [0.75, 1.20]). This ranking mirrors the distribution of their RR slopes: 7.43, 2.00, 1.61, 0.46, and − 1.65%/°C, respectively (Table 1), indicating steeper risk gradients for crashes involving vehicles on other roadways, bicyclists, and pedestrians.

**Table 1:** Estimated relative risks (RRs), RR slopes, and attributable fractions (AFs) across crash subgroups RRs compare the 99th temperature percentile to the minimum crash risk temperature, stratified by severity (all vs. fatal/severe)

Subgroup	All-severity			Fatal/severe		
Metric	RR	RR Slope (%/°C)	AF (%)	RR	RR Slope (%/°C)	AF (%)
**Modal involvement**						
Bicycle	1.50(1.33, 1.68)	-1.15	0.15%(0.10, 0.20)	2.31(1.59, 3.38)	2.00	0.36%(0.18, 0.49)
Pedestrian	1.17(1.06, 1.28)	-0.53	0.06%(-0.00, 0.11)	1.43(1.18, 1.74)	1.61	0.20%(0.05, 0.33)
Other motor vehicle on same roadway	1.15(1.11, 1.19)	-0.83	0.07%(0.05, 0.09)	1.23(1.06, 1.43)	0.46	0.14%(0.00, 0.27)
Other motor vehicle on other roadway	1.22(0.94, 1.58)	0.38	0.07%(-0.19, 0.28)	3.77(1.04, 13.61)	7.43	0.30%(-1.15, 0.84)
Fixed object	1.10(1.01, 1.19)	0.77	0.07%(0.01, 0.13)	0.95(0.75, 1.20)	-1.65	-0.09%(-0.39, 0.13)
**Collision type**						
Multi-vehicle	1.17(1.14, 1.21)	-0.84	0.08%(0.06, 0.10)	1.33(1.16, 1.52)	0.35	0.18%(0.06, 0.28)
Single-vehicle	1.15(1.07, 1.24)	0.87	0.10%(0.04, 0.16)	1.07(0.87, 1.32)	-0.62	0.02%(-0.20, 0.21)
Vehicle-pedestrian	1.19(1.08, 1.31)	0.27	0.08%(0.02, 0.13)	1.43(1.17, 1.75)	1.99	0.21%(0.06, 0.35)
**Intersection**						
Yes	1.18(1.12, 1.23)	-0.33	0.09%(0.06, 0.12)	1.49(1.25, 1.77)	1.59	0.27%(0.12, 0.39)
No	1.18(1.14, 1.22)	-0.60	0.08%(0.06, 0.10)	1.25(1.11, 1.41)	0.11	0.13%(0.03, 0.22)
**Crash timing**						
Daytime	1.23(1.19, 1.27)	-0.49	0.11% (0.09, 0.13)	1.46(1.27, 1.67)	0.27	0.20% (0.10, 0.29)
Nighttime	1.00(0.95, 1.05)	-0.58	-0.00% (-0.04, 0.03)	1.15(1.00, 1.32)	0.88	0.11% (-0.02, 0.23)
**At-fault party**						
Bicyclist at fault	1.47(1.27, 1.70)	-2.28	0.13%(0.05, 0.20)	2.25(1.43, 3.55)	2.07	0.34%(0.06, 0.53)
Pedestrian at fault	1.27(1.09, 1.48)	-0.08	0.08%(0.06, 0.11)	1.39(1.08, 1.79)	1.03	0.16%(0.06, 0.26)
Motor vehicle at fault	1.16(1.13, 1.20)	-0.34	0.17%(0.06, 0.27)	1.25(1.11, 1.40)	0.69	0.24%(-0.02, 0.45)
**Violation type**						
Pedestrian violation	1.34(1.15, 1.56)	0.75	0.17%(0.06, 0.27)	1.50(1.16, 1.93)	1.74	0.24%(-0.02, 0.45)
Driver-related violation	1.17(1.14, 1.21)	-0.52	0.08%(0.06, 0.10)	1.26(1.13, 1.41)	0.46	0.15%(0.06, 0.23)

RR slopes indicate the percent change in log(RR) per 1 °C between the 95th and 99th percentiles. AFs represent the proportion of crashes attributable to temperatures at or above the 99th percentile

**Fig. 3:**
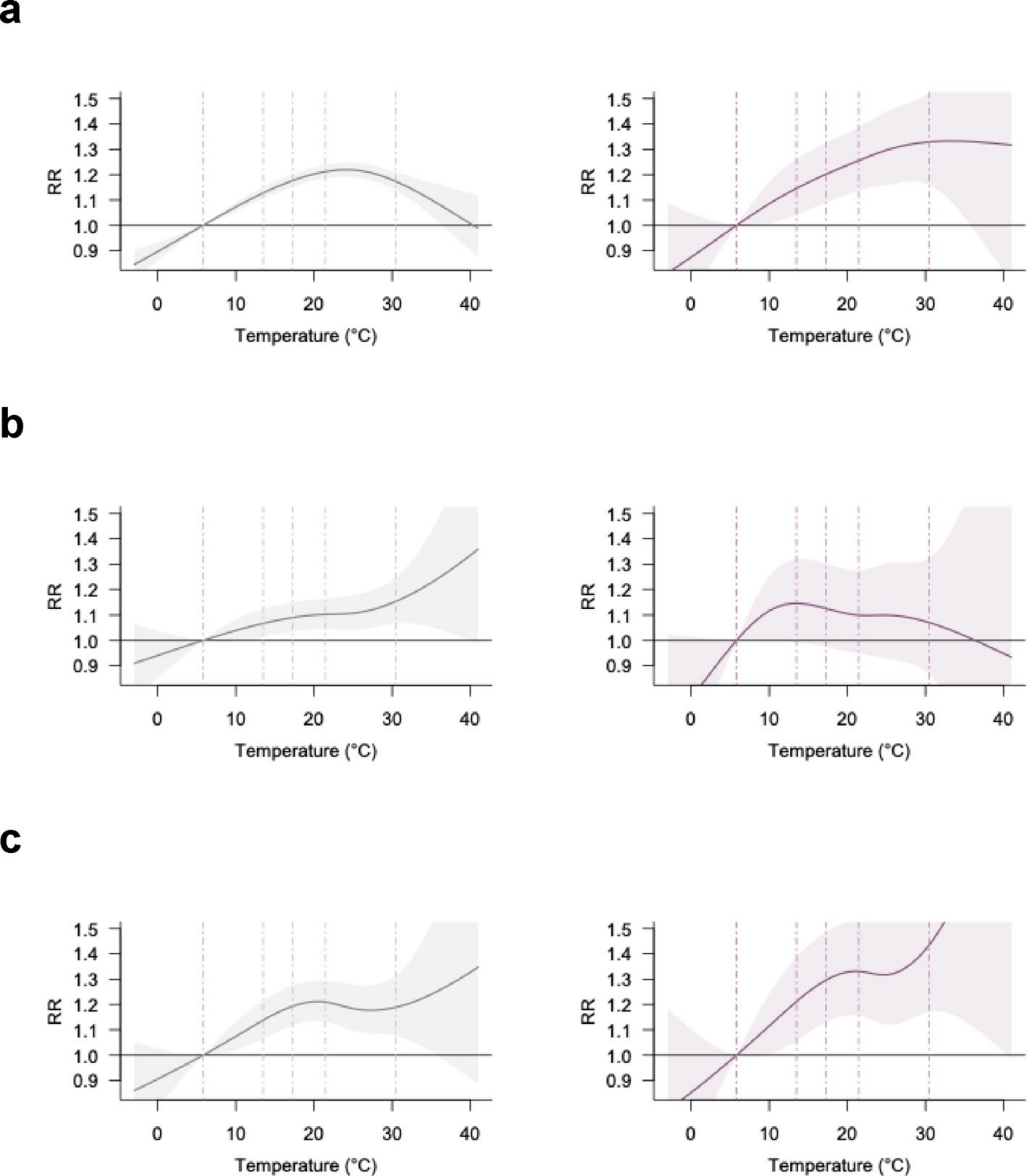
Temperature-crash associations by crash type. **a**, multi-vehicle; **b**, single-vehicle; **c**, vehicle-pedestrian. Left and right panel are all-severity and fatal and severe crash-specific analysis, respectively. Dashed lines indicate the 1st, 25th, 50th, 75th, and 99th percentiles of the temperature distribution

Distinct temperature–crash risk patterns also emerged across different crash types. In fatal/severe crashes, multi-vehicle crashes exhibited a continued increase in risk (Fig. 3a). Further disaggregation shows that the effects of extreme heat on crash risks for broadside crashes were the highest among all four subcategories of multi-vehicle crashes (Fig. S3). Single-vehicle crashes showed a continued increase in risk across all severity levels (Fig. 3b, left). In contrast, vehicle-to-pedestrian crashes followed a unique pattern: risk initially rose, plateaued around the 80th temperature percentile, and rose again at extreme heat levels (Fig. 3c). RRs increased from all-severity to fatal/severe crashes for both multi-vehicle and vehicle–pedestrian collisions, accompanied by steeper RR slopes, particularly for vehicle–pedestrian crashes (RR = 1.43 [1.17, 1.75]; RR slope = 1.99).

**Fig. 4:**
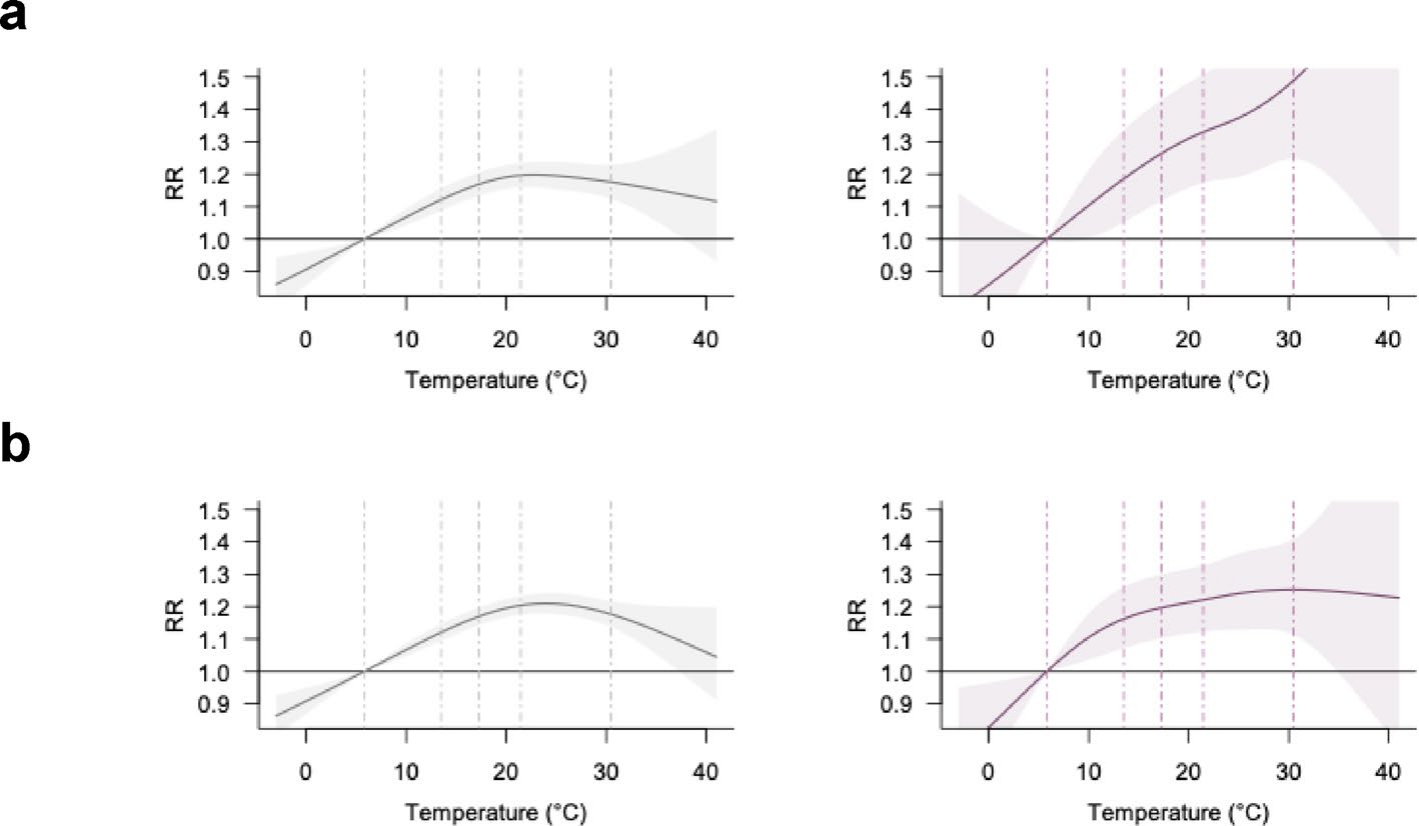
Temperature-crash associations by intersection occurrence. **a**, intersection; **b**, non-intersection. Left and right panel represent all-severity and fatal and severe crash-specific analysis, respectively. Dashed lines indicate the 1st, 25th, 50th, 75th, and 99th percentiles of the temperature distribution

Crash risk at intersections appeared more sensitive to extreme heat, particularly for fatal/severe outcomes (Fig. 4). In fatal/severe crashes, risks rose sharply at intersection locations with higher temperatures but plateaued at non-intersections. While RRs were similar across intersection types for all-severity crashes, differences widened for fatal/severe crashes. RR slope at extreme heat underscores this contrast (Table 1)—steeper at intersections (1.59%/°C) versus nearly flat at non-intersections (0.11%/°C).

**Fig. 5:**
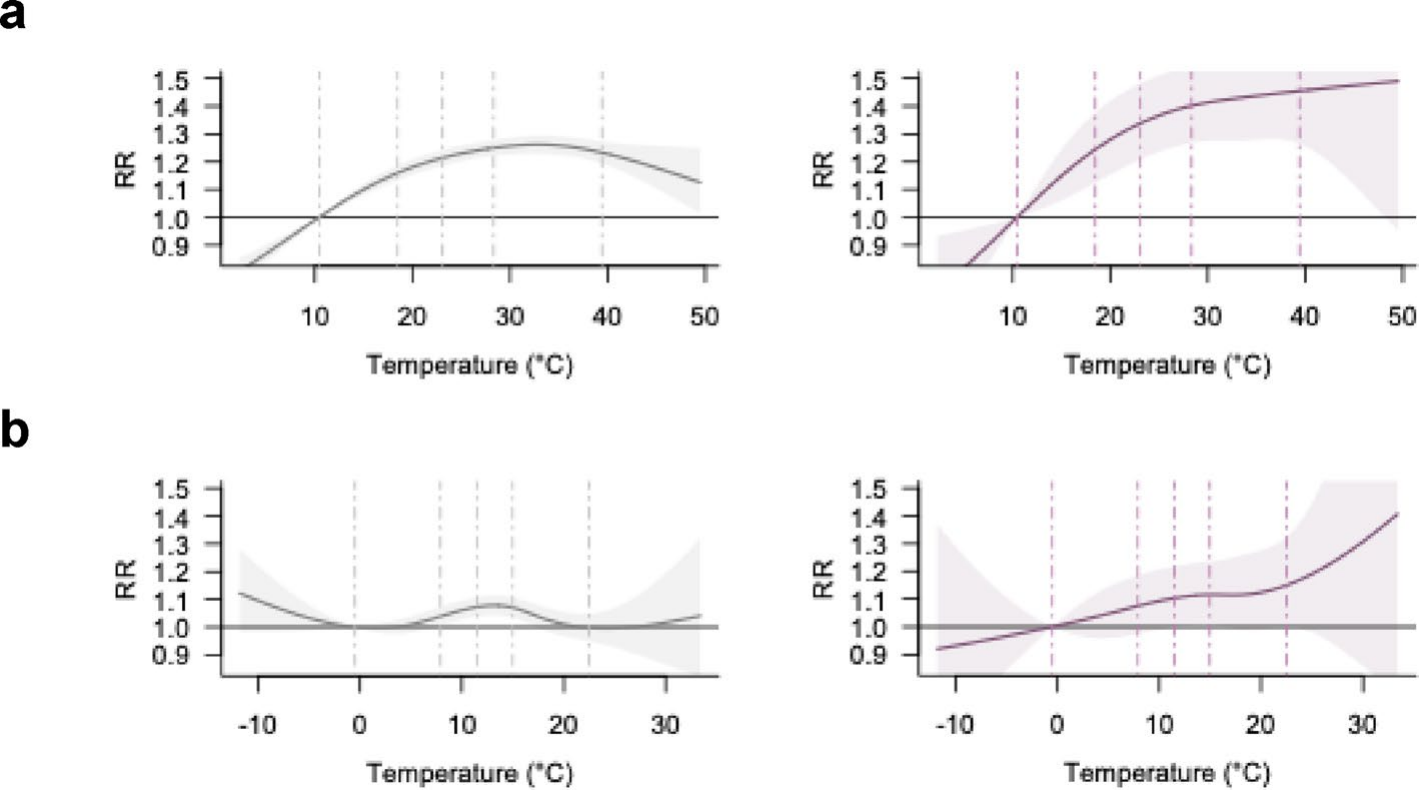
Temperature-crash associations by crash timing (solar time). **a**, Daytime; **b**, Nighttime. Left and right panel represent all-severity and fatal and severe crash-specific analysis, respectively. Dashed lines indicate the 1st, 25th, 50th, 75th, and 99th percentiles of the temperature distribution

Stratification by crash time revealed distinct patterns for daytime and nighttime crashes (Fig. 5). Daytime crashes showed an inverted U-shaped association for all-severity outcomes, whereas for fatal/severe outcomes both daytime and nighttime crashes exhibited increasing risk at higher temperatures. Although the RR for fatal/severe crashes was higher during the daytime (1.46 [1.27, 1.67]) than at nighttime (1.15 [1.00, 1.32]), the temperature–risk gradient diverged at extreme heat: the RR slope was weaker during the day (0.27%/°C) but steeper at night (0.88%/°C), indicating a greater increase in risk under extreme nighttime heat. This pattern was consistent under both solar-time and clock-time classifications (Fig. S4).

**Fig. 6:**
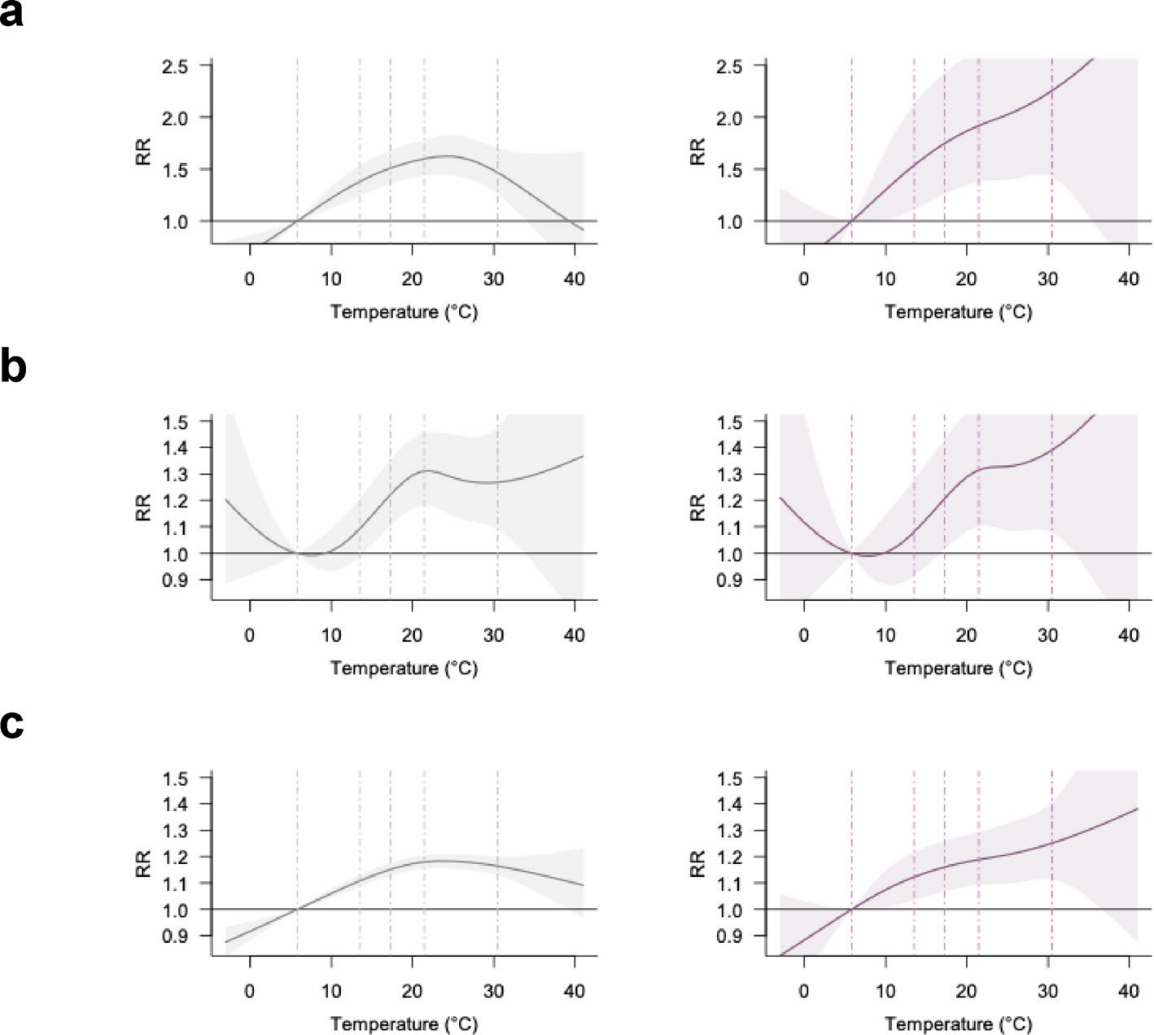
Temperature-crash associations by at-fault party’s transportation mode. **a**, bicycle; **b**, pedestrian; **c**, motorist. Left and right panel represent all-severity and fatal and severe crash-specific analysis, respectively. Note that the y-axis limits for subfigure b are adjusted to accommodate full curves. Dashed lines indicate the 1st, 25th, 50th, 75th, and 99th percentiles of the temperature distribution

Heat-related crash risk also varied by the transportation mode used by the at-fault party: heat effects were strongest for bicyclists (Fig. 6a, note the different y-axis scale), followed by pedestrians (Fig. 6b) and motorists (Fig. 6c). In fatal/severe crashes, RRs were highest for bicycle-at-fault cases (2.25 [1.43, 3.55]), compared to pedestrian- (1.39 [1.08, 1.79]) and motorist-at-fault crashes (1.25 [1.11, 1.40]) (Table 1). This marks a substantial increase from the all-severity estimate for bicycle-at-fault crashes (RR = 1.47 [1.27, 1.70]), greater than the corresponding changes seen in the other groups. Similarly, RR slopes steepened more sharply for bicycle-at-fault crashes—from − 2.28%/°C (all-severity) to 2.07%/°C (fatal/severe)—while changes for pedestrians (–0.08 to 1.03%/°C) and motorists (–0.34 to 0.69%/°C) were more modest (Table 1).

**Fig. 7:**
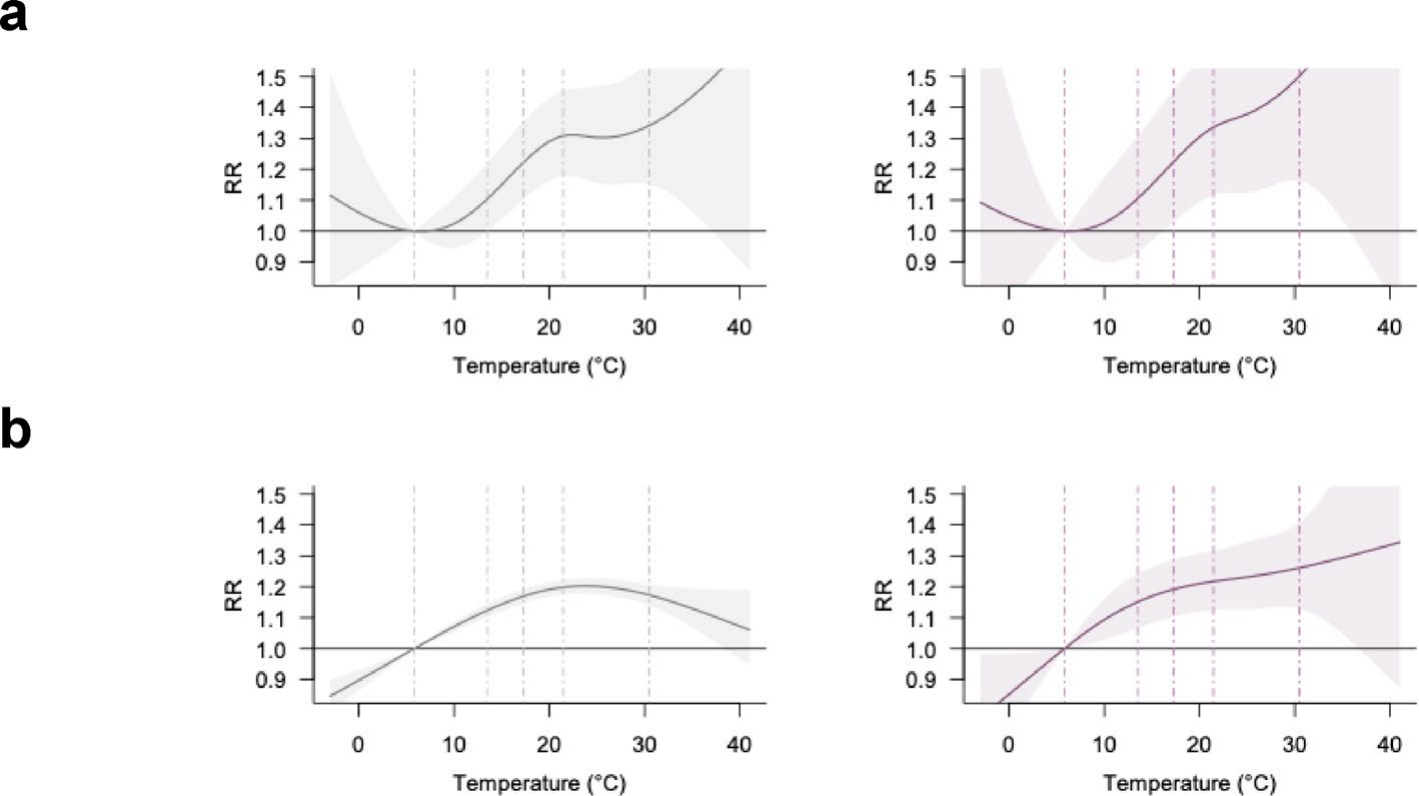
Temperature-crash associations by violation type. **a**, pedestrian violation; **b**, driver-related improper driving. Left and right panel are all-severity and fatal and severe crash-specific analysis, respectively. Dashed lines indicate the 1st, 25th, 50th, 75th, and 99th percentiles of the temperature distribution

Finally, heat-related risks varied by violation type, with pedestrian violations showing stronger associations with extreme heat (Fig. 7). Crashes involving pedestrian violations increased steadily with temperature in both all-severity and fatal/severe crashes, with RRs rising from 1.34 [1.15, 1.56] to 1.50 [1.16, 1.93], and RR slopes from 0.75%/°C to 1.74%/°C. In contrast, driver-related violations followed an inverted U-shaped pattern in all-severity crashes (RR = 1.17 [1.14, 1.21]; slope = − 0.52%/°C), shifting to a modest monotonic increase in fatal/severe crashes (RR = 1.26 [1.13, 1.41]; slope = 0.46%/°C). Further disaggregation (Fig. S5) reveals that specific driver violations—particularly improper turning and failure to yield to pedestrians—were more sensitive to heat.

## Discussion

Our study identifies nuanced and heterogeneous associations between heat and crash risks in 177 California cities, with distinct patterns emerging by injury severity, modal involvement, intersection occurrence, crash time, and at-fault characteristics. The findings indicate that heat-related risks are not uniformly distributed but appear more pronounced for high-severity crashes, particularly in high-conflict settings such as intersections and cross-traffic environments; among vulnerable road users, including bicyclists and pedestrians; and in crashes involving violations common to these contexts. These patterns deepen our understanding of how extreme heat is associated with variations in crash occurrence and severity and are suggestive of behavioral and contextual processes.

### Overall pattern

Overall, crash risks followed an inverted U-shaped pattern for all severities, while high-severity crashes showed a steady increase rose steadily with temperature, with sharper rises (steeper RR slopes) at the extreme heat end. This divergence suggests that while moderate heat may generally increase the likelihood of all crashes, extreme heat is disproportionately associated with more severe outcomes. The inverted U-shape for all severities may reflect behavioral adaptations, such as reduced travel or increased caution at temperature extremes [47, 48], which may reduce the likelihood of lower-severity crashes. However, these adaptations may be insufficient to offset processes associated with high-severity crashes under extreme heat.

In addition, the observed increase in fatal and severe crash risk at higher temperatures is consistent with findings from Spain [21], Taiwan [13], and a recent multi-city study across Latin America [45]. Notably, as Latin America is generally characterized by warmer climatic conditions, that study reported stronger and more consistent monotonic heat-related gradients in warmer cities, while non-monotonic (U-shaped) associations were primarily observed in colder cities where temperature distributions crossed the freezing point. These patterns suggest that in relatively warmer regions such as California, where sub-zero conditions are rare, heat may represent the more prominent climatic concern for severe crash risk. While fatal/severe crashes constitute only ~ 7% of our sample, their outsized societal costs underscore the urgency of integrating climate resilience into road safety strategies, particularly as rising temperatures may stall progress toward global crash reduction goals set by SDGs 3.6 and Vision Zero.

### Differential risks across road users

Fatal and severe crashes involving non-motorized road users were more strongly associated with extreme heat than those involving motorists. These findings are consistent with previous findings [13, 14] and broader evidence that vulnerable road user injury risks are sensitive to seasonal contexts, including elevated risks observed during summer periods than winter periods [49, 50]. This pattern may reflect a combination of exposure-related and vulnerability-related mechanisms.

First, from an exposure perspective, pedestrians and bicyclists are directly and unshieldedly exposed to ambient heat, unlike occupants of enclosed vehicles. Although speculative, extreme heat may also alter macroscopic travel behavior: on hot days, more individuals may shift to climate-controlled modes, increasing automobile traffic [51], while cycling [52] and walking [53] decline. This can weaken the “safety in numbers” effect, raising per capita risk for those still using non-motorized modes [54]. With fewer pedestrians and bicyclists to temper driver behavior, vehicle speeds may rise, intensifying collision severity. These altered road dynamics may disproportionately burden those who are unable to avoid travel during extreme heat and must remain mobile, often socioeconomically disadvantaged individuals without access to climate-controlled vehicles.

Second, from a vulnerability perspective, non-motorized road users lack structural protection during collisions, making them more susceptible to severe outcomes. Vehicle failures associated with extreme heat such as overheating brakes, tire stress, and road failures such as buckling and greasy pavement may contribute to the decreased safety performance of motorists, with pedestrians and bicyclists carrying most of the crash injury burden. Another possible explanation is that, in addition to ambient heat exposure, active travel generates metabolic heat and is associated with physiological stress during traffic exposure [55]. This combined thermal burden may exacerbate physiological responses, including hyperthermia and impaired coagulation [56], potentially worsening trauma outcomes. Moreover, emergency medical services may be more strained during heat events due to increased demand [57], which could further influence post-crash survival. Together, these factors may increase the likelihood that crashes occurring under extreme heat result in severe or fatal outcomes.

Third, we observed that bicyclists and pedestrians were more likely to be involved in reported violations or to be assigned fault during extreme heat. This pattern may reflect that active travelers are more prone to heat-related cognitive and physiological strain, given their direct exposure and exertional load, resulting in behavioral changes, such as cognitive impairment [55], distraction [58], and slower response times [59]. Heat stress may also impair risk assessment, triggering risky road behaviors under extreme heat, such as red-light running by bicyclists [60] or mid-block crossings by pedestrians [61]. Though pedestrians and bicyclists are less likely to be at fault in typical crashes [62], extreme heat may alter these dynamics, influencing behavioral patterns in ways that compound risk and liability for non-motorized road users.

### Differential risks across crash contexts and plausible mechanisms

Our findings also shed light on plausible mechanisms through which heat may be associated with elevated crash risks, particularly behaviors captured in the collision data, such as traffic violations and the types of collisions. While our dataset does not allow for direct assessment of mechanical or infrastructural pathways, future linkage with other data sources may enable such analyses. Notably, we found higher risks in traffic scenarios demanding heightened attention and rapid decision-making. For example, the stronger effect of extreme heat on intersection crashes is likely driven by conflicting traffic flows and the frequent presence of pedestrians and bicyclists. Broadside crashes—commonly occurring at intersections due to cross-traffic movements—were more sensitive to extreme heat, highlighting the susceptibility of these high-conflict environments during high-temperature conditions.

Heat was also more strongly associated with increased risk from improper turning and failure to yield to pedestrians. Collisions involving vehicles from different roadways (e.g., merging or crossing from adjacent roads) showed higher heat-related risk than those involving vehicles traveling on the same roadway, likely due to greater unpredictability and decision-making complexity—factors likely amplified by heat-induced distraction or impaired judgment.

While prior studies have linked heat-related crashes to mechanical issues (e.g., engine overheating) or infrastructural degradation (e.g., buckled pavement) [23, 24], our findings may suggest these are unlikely to be the sole or dominant mechanisms. If infrastructural factors were the primary mechanism, we would expect similar effects between intersection and non-intersection crashes, as degraded road conditions would plausibly affect both intersections and straight road segments alike. Likewise, if mechanical issues like overheating were the main cause, risks would likely decline more at night when temperatures drop below critical failure thresholds, though residual heat from the day may still impair vehicle function.

### Limitations and future directions

Several limitations are noted. First, the use of police-reported crash data might introduce reporting inconsistencies, as the classification of crash severity, event-causing violations, and fault assignment is subject to the discretion of the responding officer. While severity coding in SWITRS follows MMUCC guidelines, some degree of misclassification is possible, particularly in distinguishing between minor and severe injuries [63]. Second, crashes without reported injuries were not recorded in the reporting system, limiting our ability to examine the full spectrum of crash outcomes. Third, we did not account for meteorological factors such as humidity or wind speed, which may influence the physiological stress associated with heat exposure. Fourth, daily traffic volume data (e.g., vehicle-miles traveled, VMT) were unavailable, which is a common limitation in large-scale road safety studies. Yet, our time-stratified case-crossover design helps mitigate this limitation by matching case days to control days on the same day of the week within the same city, month, and year, thereby controlling for recurring traffic patterns and seasonal trends. Prior methodological work has demonstrated that such designs approximate baseline traffic volume patterns [19] and yield effect estimates comparable to models that explicitly adjust for traffic volume measures [40], supporting the validity of our approach. In addition, although traffic volume may plausibly mediate any temperature effects on crashes—for example, extreme heat or cold could alter travel volumes and indirectly influence crash risk—our research design allows us to estimate the overall short-term association between heat and crash occurrence rather than aiming to decompose indirect pathways through changes in traffic exposure. Nonetheless, future research incorporating direct traffic counts or VMT data would help clarify the potential mediating role of traffic exposure and other factors, thereby further strengthening the interpretation of these findings. Lastly, although our study spans 177 cities, statistical power was limited for rare outcomes. We therefore pooled data to estimate overall associations and did not examine city-specific variations, such as differences in roadway design and enforcement practices, given the already wide confidence intervals observed for fatal and severe crashes. Additional stratification for rare strata, including crashes involving vehicles on other roadways, yielded wide confidence intervals due to limited event counts, reflecting reduced statistical power. Accordingly, these estimates should be interpreted cautiously and regarded as hypothesis-generating rather than definitive, pending confirmation in larger or pooled datasets.

Future research can build upon our findings by addressing these limitations. Additionally, further work can test the external validity of our findings in other transportation contexts. In U.S. cities, automobile-dominated road networks contribute to the severity of crashes involving vulnerable road users lacking protective enclosures, likely differing from cities with more balanced modal shares or bicyclist-friendly infrastructure, such as Amsterdam, Tokyo, and Bogotá, where higher volumes of non-motorized users may mitigate risks. Another promising research avenue involves exploring behavioral adaptations to extreme heat, such as heat-induced changes in travel patterns (e.g., trip making and mode switching), risk perception, and road behavior.

## Conclusion

This study offers new insights into heat-related crash risks, demonstrating how these risks vary significantly by crash-level context. We highlight that extreme heat disproportionately increases the risk of higher-severity crashes, especially at high-conflict settings and among non-motorized, vulnerable road users. By integrating detailed police-reported crash records with high-resolution temperature data across 177 California cities over more than a decade, our approach enables a granular yet scalable analysis that advances beyond prior work typically focused on aggregate crash counts. This yields two key empirical contributions: first, we offer evidence supporting behavioral explanations to heat-induced crash risk; second, we show extreme heat may alter traditional crash dynamics by increasing fault attribution to pedestrians and bicyclists.

Our findings carry several practical implications for road safety and climate adaptation. Most directly supported by our results, the elevated risks observed during extreme heat, particularly for pedestrians, cyclists, intersection crashes, and violation-related events, suggest that interventions targeting high-conflict urban settings should be prioritized. These may include enhanced right-of-way enforcement at intersections, targeted education on safe road behavior during heat events, and the provision of shaded or cooled infrastructure along walking and cycling corridors. Because crash risks intensified rapidly during hot periods, timely public heat advisories for road users may also serve as a preventive strategy. More broadly, reducing overall driving by expanding public transportation and supporting active travel for short trips could further mitigate population exposure to both heat and crash risks. At an institutional level, heat should also be formally recognized as a situational risk factor within police crash reporting forms and safety surveillance systems. Current reporting frameworks typically capture weather conditions that impair visibility or vehicle handling (e.g., snow, fog, or high winds), but do not explicitly document high-temperature conditions, limiting the ability of researchers and agencies to systematically track and evaluate heat-related crash risks. Together, these findings underscore the need to better integrate climate adaptation and road-safety planning. As cities face rising temperatures, extreme heat should be considered not only a public-health concern but also a transportation-safety priority.

## Data Availability

All data used in this study are publicly available. Temperature data is available from [PRISM Climate Group](https:/prism.oregonstate.edu) . Road crash data is available from the Statewide Integrated Traffic Records System (SWITRS), maintained by the California Highway Patrol and accessible through the [Transportation Injury Mapping System](https:/tims.berkeley.edu/help/SWITRS.php) developed by UC Berkeley’s Safe Transportation Research & Education Center (SafeTREC). Population estimates used for temperature weighting are available from the [U.S. Census Tract Population Data](https:/seer.cancer.gov/censustract-pops) provided by the National Institutes of Health.

## Code availability

The code repository is available at https://github.com/chengkaihsu/CCUH_CA.
